# 1515. Implementing “Opt-Out” Screening to Identify and Link Patients to Care With HIV: Experience from Memorial Healthcare System

**DOI:** 10.1093/ofid/ofad500.1350

**Published:** 2023-11-27

**Authors:** Paula A Eckardt, Jianli Niu, Elizabeth J Piche

**Affiliations:** Memorial Healthcare System, Hollywood, Florida; Memorial Healthcare System, Hollywood, Florida; Memorial Healthcare System, Hollywood, Florida

## Abstract

**Background:**

The program is an opt-out HIV testing program aiming to improve HIV screening and provide linkage to care for patients who test positive. Here we present project results of implementing opt-out” screening to identify and link patients to care with HIV in a large community hospital in South Florida.

**Methods:**

Patients aged 18-75 years entering the emergency departments (EDs) for medical care were screened for eligibility automatically utilizing an EPIC-based screening tool. The program data from its inception in June 2008 to the end of December 2022 were retrospectively reviewed. Data included demographics and HIV testing results with linkage to care information. All HIV positive results were confirmed by Broward County Department of Health to identify new versus previous HIV positive cases. Data was entered in a spreadsheet and then imported for descriptive statistical analysis.

**Results:**

Of the 79,309 patients tested in the program between June 2018 and December 2022, 471 were identified as HIV positive with an overall rate of 0.6% (Figure 1). Of those tested HIV-positive, 134 (28.5%) were newly diagnosed. The majority of those newly diagnosed were male (64%), African American (66%), and 26-35 years of age (27.2%). 74.6% of the newly diagnosed patients were linked to care (Figure 2). Of those 337 previously diagnosed patients, 189 (56.1%) were confirmed in HIV care, 20 were known to have died, and 68 (53.1%) of 128 patients out of care at testing achieved care re-engagement (Figure 2). Additionally, the number of opt-out tests decreased from 22,092 in 2019 to just 10,359 in 2020 due to the COVID-19 pandemic. However, as the pandemic subsided in 2021 and 2022, the number of opt-out tests increased to 14,705 and 22,264 respectively (Figure 1).

**Figure 1.**
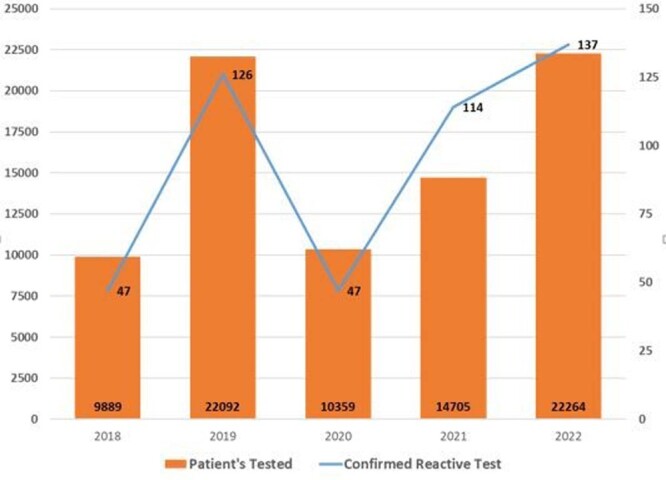
The FOCUS testing HIV data at MHS, June 2018−December 2022.

**Figure 2.**
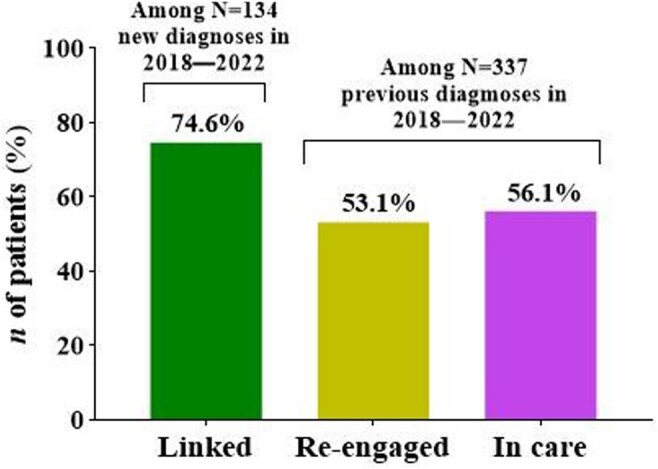
Linkage to medical care in HIV-positive cases, June 2018−2022.

**Conclusion:**

The results showed that the FOCUS program on HIV testing and linkage to care can be successfully integrated into a community healthcare system. The high rate of newly diagnosed HIV-positive cases in the program highlights the need for increased access to HIV testing and healthcare services. The rate of HIV re-engaged in care among those previously diagnosed also indicates the need for opt-out HIV testing in the community to increase reengagement in care.

**Disclosures:**

**All Authors**: No reported disclosures

